# Value of ambroxol in the treatment of asthmatic bronchitis

**DOI:** 10.12669/pjms.36.3.1607

**Published:** 2020

**Authors:** Xiaoxia Du, Chengyan Zhao, Shiqing Liu, Shufen Su

**Affiliations:** 1 Xiaoxia Du, Health Department of Respiration and Neurology, The First Affiliated Hospital of Shandong First Medical University (Shandong Provincial Qianfoshan Hospital), Shandong, 250014, China; 2 Chengyan Zhao, Department of Pediatrics, The First Affiliated Hospital of Shandong First Medical University (Shandong Provincial Qianfoshan Hospital), Shandong, 250014, China; 3 Shiqing Liu, Health Department of Respiration, The First Affiliated Hospital of Shandong First Medical University (Shandong Provincial Qianfoshan Hospital), Shandong, 250014, China; 4 Shufen Su, Department of Endoscopic-Surgery, The First Affiliated Hospital of Shandong First Medical University (Shandong Provincial Qianfoshan Hospital), Shandong, 250014, China

**Keywords:** Ambroxol, Therapeutic effect, Asthmatic bronchitis

## Abstract

**Objective:**

To investigate the clinical effect of ambroxol in the treatment of asthmatic bronchitis.

**Methods:**

One hundred and twenty patients with asthmatic bronchitis who were admitted to our hospital from June 2017 to August 2018 were selected as the research subjects and divided into a control group and an observation group according to random number table method, 60 in each group. The control group was treated with conventional treatment, while the observation group was treated with ambroxol in addition to conventional treatment. The therapeutic effect, disappearance time of symptoms and signs and the recovery of pulmonary function were compared between the two groups.

**Results:**

The total effective rate of the observation group was 96.7%, and that of the control group was 73.3%. The control effect of the observation group was significantly better than that of the control group, showing a significant difference (P<0.05). The disappearance time of symptoms of the observation group was shorter than that of the control group, and the recovery of pulmonary function was better; the differences were statistically significant (P<0.05).

**Conclusion:**

For asthmatic bronchitis patients, addition of ambroxol to conventional treatment can improve the therapeutic effect, shorten the disappearance time of clinical signs and symptoms, and promote the recovery of patients, which is worth clinical application.

## INTRODUCTION

Asthmatic bronchitis is chronic airway inflammation. The joint participation of T lymphocyte, mastocyte and eosnophils is the pathogenesis of asthmatic bronchitis. The main symptoms of asthmatic bronchitis are asthma, cough, dyspnea, phlegm and lung wheezing.^1^,^2^ It has a serious impact on the physical and mental health of patients; therefore, early diagnosis and treatment is needed. The etiology of asthmatic bronchitis has not been clarified yet. Some researchers advocate that it is related to factors such as heredity and environment and it is prone to recurrence. If the treatment is not timely, it can evolve into bronchial asthma, and the difficulty of treatment will greatly increase.^3^,^4^

Asthmatic bronchitis has a slow onset and a long course. Repeated acute attacks can aggravate disease condition, which may lead to many complications such as emphysema, pneumonia and pulmonary heart disease.^5^ It is reported that asthmatic bronchitis patients often havediseases such as eczema and allergy. After being attacked by pathogens, infection and inflammation may occur, resulting in local congestion of airway, increased secretions and airway stenosis. Therefore, it is necessary to reduce airway secretions and expel sputum to control airway inflammation and achieve the goal of treating asthmatic bronchitis.^6^,^7^ At present, the treatment methods mainly include oxygen inhalation, cough relief, asthma relief, spasmolysis, anti-infection, aerosol inhalation, etc., which can relax airway smooth muscle to improve symptoms; but the above-mentioned treatment methods cannot effectively achieve the goal of phlegm resolving and expectoration and cannot fundamentally solve the problem of respiratory tract dyspnea.^8^

Ambroxol, a kind of phlegm dissolving agent, can stimulate the secretion of active agent adhered to the surface of the respiratory tract. Moreover, it can regulate the serous secretion and mucinous secretion. A foreign study pointed out that ambroxol had a significant effect in preventing bronchitis.^9^ However, reports on the clinical application of ambroxol in the treatment of asthmatic bronchitis are few; therefore, this study selected 120 patients with asthmatic bronchitis who were admitted to our hospital from June 2017 to August 2018 as the research subjects to explore the application effect of ambroxol in the treatment of asthmatic bronchitis.

## METHODS

One hundred and twenty patients with asthmatic bronchitis who were admitted to our hospital from June 2017 to August 2018 were selected as the research subjects. All patients were divided into an observation group and a control group according to random number table method, 60 each group. All the subjects in this study were diagnosed as asthmatic bronchitis by pulmonary function examination. The results of the examination were in accordance with the international diagnostic criteria of asthmatic bronchitis.^10^ Patients who had severe liver and kidney dysfunction, severe endocrine disorders, severe metabolic diseases, a history of drug allergy or severe cancer diseases were excluded. All patients and their families were informed about the study and signed informed consent. This study has been approved by the hospital ethics committee. (Nov. 1, 2019)

### Therapeutic Method

After admission, patients received conventional treatment of asthmatic bronchitis, i.e., including symptomatic treatment such as oxygen inhalation, cough relief and asthma relief and antibiotics for anti-infective treatment. In addition to the above treatment; ambroxol was used as adjuvant therapy in the observation group. Yinuoshu (Ambroxol Hydrochloride Injection, Tianjin Pharmaceutical Research Institute Pharmaceutical Industry Co., Ltd., SFDA approval number: H20051604) was administered by slow intravenous injection, 15 mg/time for adults, once in the morning and once in the evening. The drug administration lasted for one week.

During the treatment, the two groups cooperated with nursing staff. The specific measures were as follows. First was psychological nursing. Nurses cared for and sympathized with patients, mastered their unhealthy psychological state, and gave them comfort and encouragement to eliminate inferiority and negative psychology, throw away the burden of thought, face diseases and life correctly, enhance confidence in overcoming diseases, and improve treatment compliance. Next was conventional nursing. Besides the treatment according to doctor’s advice, daily life nursing and health education were given. Patients were instructed to quit smoking firmly and strengthen physical exercise; eat more high-quality protein, limit the intake of sodium salt and seafood, and avoid contact with pollen, fur, mold and dust.

### Observation indexes

The effect of the treatment was evaluated by improvement of clinical symptoms and pulmonary function. If the clinical symptoms such as cough and chest pain disappeared after treatment, the body temperature recovered to normal, and the blood routine and pulmonary function examination results were normal, then the disease was evaluated as cured. If the clinical symptoms and body temperature had remission and the blood routine and pulmonary function examination results improved, then the treatment was evaluated as effective. If the clinical symptoms, body temperature and pulmonary function had no remission, then the treatment was evaluated as ineffective. The calculation formula was: total effective rate= cured rate+effective rate.

(1) The improvement time of clinical symptoms was measured and recorded.
(2) The recovery of pulmonary function was compared between the two groups: before and after treatment, the forced expiratory volume in 1 second (FEV_1_) and forced vital capacity (FVC) of the two groups were monitored, and the ratio of them (FEV_1_/FVC) was calculated.

### Statistical Analysis

SPSS ver. 23.0 was used to process the data. The measurement data were expressed as Mean±Standard Deviation, and t test was used; the counting data were expressed as rate (%) and X^2^ test was used. P<0.05 indicated that there was significant difference.

## RESULTS

There were 26 males and 34 females in the observation group, aged from 21 to 44 years, with an average age of (32.5±11.5) years. The course of disease ranged from one to six years, with an average disease course of (2.6±1.7) years. In the control group, there were 28 males and 32 females, aged from 23 to 42 years, with an average age of (32.5±9.5) years. The course of diseases ranged from one to five years, with an average age of (2.4±1.6) years. There was no significant difference in baseline data between the two groups; therefore, the results were comparable.

***:*** The total effective rate of the observation group and control group was 96.7% and 73.3% respectively. The treatment effect of the observation group was significantly better than that of the control group, and the difference between the two groups in the treatment effect had statistical significance (P<0.05, Table-I).

**Table-I:** Clinical efficacy between the two groups [n (%)].

Group	Cured	Effective	Ineffective	Total effective rate
Observation group	54(90)	4(6.7)	2(3.3)	58(96.7)
Control group	33(55)	11(18.3)	16(26.7)	44(73.3)
X^2^	/	/	/	11.749
P	/	/	/	<0.05

The disappearance time of symptoms and signs in the observation group was significantly shorter than that in the control group, and the difference had statistical significance (P<0.05, Table-II).

**Table-II:** Disappearance time of symptoms and signs between the two groups.

Group	Disappearance time of asthma	Disappearance time of wheezing	Disappearance time of cough	Disappearance time of rale
Observation group	3.06±0.82	3.12±0.78	5.10±2.85	2.12±1.36
Control group	5.46±1.83	5.71±1.62	7.82±2.91	4.68±1.59
t	8.357	10.185	4.701	8.618
P	<0.05	<0.05	<0.05	<0.05

The pulmonary function in the two groups improved after treatment compared with that before treatment. The improvement degree in the observation group was significantly better than that in the control group, and there was a significant difference between the two groups (P<0.05, Table-III).

**Table-III:** Pulmonary function indexes between the two groups before and after treatment.

Group		FEV_1_ (L)	FEV_1_/FVC (%)
Observation group	Before treatment	1.31±0.36	34.57±0.62
	After treatment	2.47±0.49	65.83±1.27
Control group	Before treatment	1.28±0.38	34.49±0.58
	After treatment	1.85±0.42	54.66±1.52

## DISCUSSION

Asthmatic bronchitis is a chronic airway inflammation, not belonging to asthma, but there are still some links between them, as some cases of asthmatic bronchitis will develop into asthma over time.^11^ As to the etiology of asthmatic bronchitis, there are two main factors, one is genetic factor. Relevant studies have shown that multi-locus genes are associated with allergic diseases, which plays an important role in the pathogenesis of asthma.^12^ Secondly, environmental factors such as dust, gasoline, smoke, paint and other irritating odors and cold air can stimulate the bronchial mucosa and cause vagal nerve excitation and cough.^13^ It was found that the degree of pulmonary function damage was gradually aggravated with the prolongation of the course of asthmatic bronchitis.^14^ Without timely and effective treatment in clinic, the patients will have complications of cardiopulmonary failure, which will seriously endanger people’s quality of life.

Ambroxol is a new type of expectorant, which can regulate mucus viscosity, dilute mucus, clean up oxygen free radicals, promote the synthesis of surfactant in alveoli, strengthen ciliary movement, enhance the clearance function of mucus transport system, and dissolve and expel sputum to improve the respiratory tract ventilation function of patients and make patients recover as soon as possible.^15^,^16^ Moreover, ambroxol cannot only inhibit the release of inflammatory transmitters, such as mast cells, basophils and so on, to avoid lung infection and damage, but also inhibit histamine-induced contraction of airway smooth muscle to relieve cough.^17^ Ambroxol can be absorbed better if being intravenously injected than being orally administrated, and its bioavailability is up to 90%. It has the characteristic of long-lasting pharmacodynamics. After liver metabolism, inactive dibromo-o-aminobenzoic acid can be formed, 80% of which is metabolized through the kidney prototype.^18^ The application of ambroxol in patients with asthmatic bronchitis is significantly effective in promoting the dissolution of sputum and the increase of blood concentration. In this study, ambroxol was used to treat patients with asthmatic bronchitis. The results showed that the effective rate was 96.7% in the observation group and 73.3% in the control group, and the difference was statistically significant (P<0.05); the overall effect of the observation group was better, which was consistent with the results of previous studies.^19^,^20^

Zhang et al. found that the use of ambroxol combined with antibiotics can increase the penetration of antibiotics to lung tissue and enhance the bactericidal ability of antibiotics.^21^ This study shows that ambroxol is more effective than conventional treatment in the treatment of asthmatic bronchitis in improving clinical symptoms and pulmonary function, which is in line with the results of Li.^22^ It can be seen that ambroxol is effective in treating asthmatic bronchitis as it can effectively control the disease condition and relieve symptoms.

## CONCLUSION

Aambroxol has a good clinical effect in the treatment of asthmatic bronchitis, and it can significantly improve symptoms such as asthma and cough, control patient’s condition, improve the quality of treatment, and promote the recovery of patients. It is worth promotion and application in clinical treatment.

### Authors’ Contribution:

**XXD & SFS:** Study design, data collection and analysis.

**XXD, CYZ, SQL & SFS:** Manuscript preparation, drafting and revising.

**SFS:** Review and final approval of manuscript.

**XXD:** Is responsible and accountable for the accuracy or integrity of the work.
